# Similar Material Proportioning Tests and Mechanical Properties Based on Orthogonal Design

**DOI:** 10.3390/ma16196439

**Published:** 2023-09-27

**Authors:** Xinglong Yang, Jinyu Dong, Jihong Yang, Xiaodong Han

**Affiliations:** College of Geosciences and Engineering, North China University of Water Resources and Electric Power, Zhengzhou 450046, China; yangxinglong@stu.ncwu.edu.cn (X.Y.); yangjihong@ncwu.edu.cn (J.Y.); hanxiaodong@stu.ncwu.edu.cn (X.H.)

**Keywords:** similar materials, orthogonal design, double-sided shear, uniaxial compression, splitting test, multiple regression analysis

## Abstract

Shaking table tests serve as an effective method to simulate landslides triggered by seismic activities. These laboratory experiments necessitate the use of materials that mimic those encountered in real-world scenarios. For this investigation, materials analogous to field conditions for the shaking table tests were formulated using quartz sand, barite powder, iron powder, gypsum, rosin, and alcohol. Within the model test compositions, iron powder, barite powder, and quartz sand acted as aggregates; gypsum functioned as an additive, and a solution of rosin and alcohol was employed as a binder. Employing the orthogonal design method, the physical and mechanical parameters of these analogous materials were ascertained through double-sided shear tests, as well as uniaxial compression and splitting tests. Subsequent analyses included extreme difference and regression assessments targeting the determinants influencing the physical and mechanical characteristics of these materials. The ultimate goal was to determine the optimal mixing ratios for the model test materials. The findings revealed that the physical and mechanical properties of analogous materials at varying ratios span a broad spectrum, fulfilling the criteria for distinct rock model experiments. A thorough examination of the factors impacting the physical and mechanical properties of these materials was undertaken, elucidating their respective influences. Based on the relative significance of each determinant on the mechanical attributes of the analogous materials, dominant factors were identified for a multiple regression analysis, from which the regression equations corresponding to the test ratios were derived.

## 1. Introduction

Earthquake-induced instability frequently occurs in high and steep slopes characterized by variable morphologies and intricate rock formations. Indoor large shaking table tests [1,2,3,4,5] provide an enhanced means of replicating the entire landslide process. However, when conducting these large shaker tests in geological engineering, considerations must include the prototype’s size and the appropriate scaling of its physical and mechanical parameters. Evaluating the scaled down physical model facilitates the understanding of the prototype’s dynamic response, deformation, and damage patterns. The use of analogous materials at this stage becomes essential.

Xu [6] emphasized that model tests constitute a crucial component of the similarity theory and significantly influence contemporary scientific investigations. Huang [7] combined physical model tests with numerical analysis to emulate tunnel excavation near a mezzanine, validating the efficacy and applicability of merging model tests with numerical simulations. Li [8] elaborated on the design, fabrication, and testing methodologies for analogous models. Utilizing an orthogonal [9] test design, Liu [10] performed numerical simulations to understand the damage characteristics of the 3306 working face’s floor slabs. By employing matrix analysis and variance analysis methods, the resultant simulations were assessed to discern the principal controlling factors’ sensitivity related to the slab’s damage depth. In a model test [11,12,13], materials mirroring the prototype’s physical and mechanical attributes are integrated into a downscaled version, following specific similarity principles, and subsequently examined in alignment with the prototype’s intended functionality.

In geotechnical studies, addressing intricate geological engineering challenges often surpasses the capabilities of traditional model tests and theoretical assessments. In such instances, physical model tests employing analogous materials post-prototype downscaling render a more precise representation of the prototype’s physical and mechanical attributes. Hence, for intricate issues that defy resolution through theoretical and numerical analytical techniques [14,15,16,17,18,19,20], analogous model testing remains indispensable.

Model experimental studies place considerable emphasis on the meticulous selection and proportioning of model-like materials. Such a selection determines the model’s accuracy in mirroring the prototype’s attributes, its amenability to processing, and the smooth progression of tests. There has been extensive scholarly research on formulating analogous materials suitable for rock masses [21,22,23,24,25,26,27,28]. For instance, Liu [14] opted for a blend of iron concentrate powder, barite powder, and fine sand powder, using gypsum as the binder and glycerol for regulation. The control indices, in this case, comprised the density, compressive strength, and elasticity modulus of the analogous materials. Influential factors underwent sensitivity evaluations via extreme difference analysis and variance analysis. Introducing a novel analogous material for geotechnical engineering, Han [22] incorporated iron concentrate powder, barite powder, fine sand powder, and a rosin alcohol solution in specific proportions. This composite material’s mechanical properties [17,18,19,20], under varied proportion conditions, underwent scrutiny through standard mechanical tests, including the uniaxial compressive strength test, pseudo triaxial test, and Brazil test. Such assessments aimed to ensure its suitability for diverse physical model test applications. Zhang et al. [27,28,29,30] proposed a mixture composed of iron ore powder, barite powder, quartz sand, gypsum powder, and a rosin alcohol solution blend. Post the analogous materials’ selection [31,32,33,34,35,36,37,38,39,40], the pivotal nature of choosing an optimal test design method becomes evident, with primary experimental design methods encompassing the full factor test, orthogonal [41,42,43,44,45,46] test design, and homogeneous test design. Notwithstanding the extensive investigations into the composition, physical, and mechanical properties of analogous materials, studies focusing on sensitivity and multiple regression analyses remain scarce. Hence, this paper endeavors to fill this research void.

Drawing upon Zhang et al.’s research [27,28,29,30] and other analogous material studies, this paper employs the orthogonal test method to devise the analogous materials’ test scheme. Indoor tests of materials in varied proportions were conducted to determine the analogous materials’ physical and mechanical parameters under different ratios. An extreme difference analysis then delved into the sensitivity and influence laws each factor exerted on these parameters.

## 2. Similar Material Proportioning Orthogonal Design Solutions

### 2.1. Orthogonal Design Methods

Orthogonal experimental design is a pivotal aspect of statistical mathematics, rooted in probabilistic mathematical statistics, technical expertise, and empirical knowledge. By utilizing standardized orthogonal tables, this method organizes experimental schemes and analyzes results, aiming to minimize the number of tests, expedite the testing phase, and swiftly identify the optimal strategy. The orthogonal testing method [41,42,43,44,45,46,47] is employed for multifactorial experiments, selecting representative points from comprehensive trials. This design is a primary fractional factorial method, renowned for its efficacy. The outcome assessed in an experiment is termed the ‘indicator’, while the variables that could influence this indicator are termed ‘factors’. The distinct conditions examined for each factor are designated as ‘levels’. An experiment is considered orthogonal if it adheres to two criteria: (i) diverse levels of each factor appear equally in the experiment (homogeneity) and (ii) varied combinations of levels from any two factors manifest uniformly (orthogonality). This approach, when juxtaposed with a comprehensive experimental design, is termed orthogonal testing. By employing an orthogonal table to structure the experiment, the distribution of testing points is even, subsequently reducing the cumulative number of tests.

### 2.2. Material Selection and Factor Setting

In the present investigation, the integration of several variables into one resulted in a marked decrease in test numbers, enhancing test efficiency without compromising result stability or precision. The materials employed included iron ore powder, barite powder, and quartz sand as aggregates, with rosin alcohol serving as the binder and gypsum as the conditioning agent. The iron ore powder, boasting a specific gravity of 4.3, was sourced from the Heilongjiang Tonghua Iron Ore Plant, Jilin City, China. Barite powder was procured from Jiaozuo Zhuangli Fine Alcohol Chemical Co., Jiaozuo, China. For the experimental framework, four primary factors were considered in the orthogonal design: the combined ratio of iron powder and barite powder to aggregate (factor A), the proportion of iron powder in the combination of iron powder and barite powder (factor B), the binder concentration defined by the ratio of rosin to the sum of rosin and alcohol (factor C), and the content of gypsum (factor D). Each factor encompassed five levels, delineated in Table 1. Table 2 illustrates the material proportioning scheme. Hereafter, the four factors are denoted as A, B, C, and D.

## 3. Preparation of Specimens and Mechanical Tests

### 3.1. Preparation of Specimens

**Figure 1:**
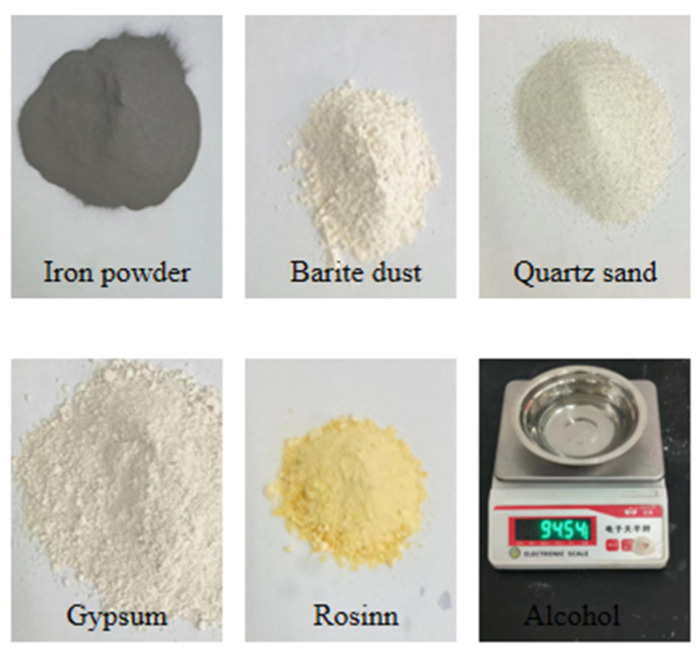
Similar materials. Non-English words on weighting instrument mean electronic balance.

**Figure 2:**
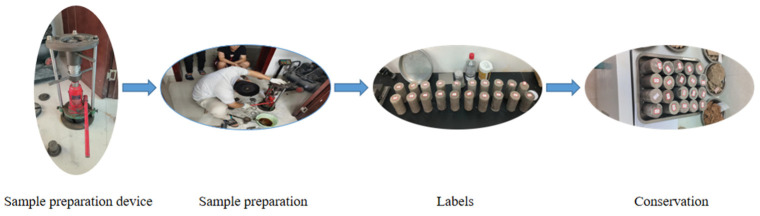
Sample production process.

Utilizing the aforementioned orthogonal design method, specimens were fashioned based on varying proportions. The core procedure for specimen preparation entailed the following steps. Initially, iron powder, barite powder, quartz sand, and gypsum were weighed to their respective proportions and subsequently mixed thoroughly. Following this, the previously prepared rosin alcohol solution was added to the mixture, ensuring a consistent blend before transferring it into the mould. The sample was then compacted using a custom-made pressure apparatus. Once compacted, the specimens were demoulded and labelled, as depicted in Figure 1 and Figure 2. The samples were air-dried under ambient conditions for a duration of 2–3 days, succeeded by the execution of the uniaxial compression test, the bilateral shear test, and the Brazilian splitting test [47].

### 3.2. Uniaxial Compression Test

**Figure 3:**
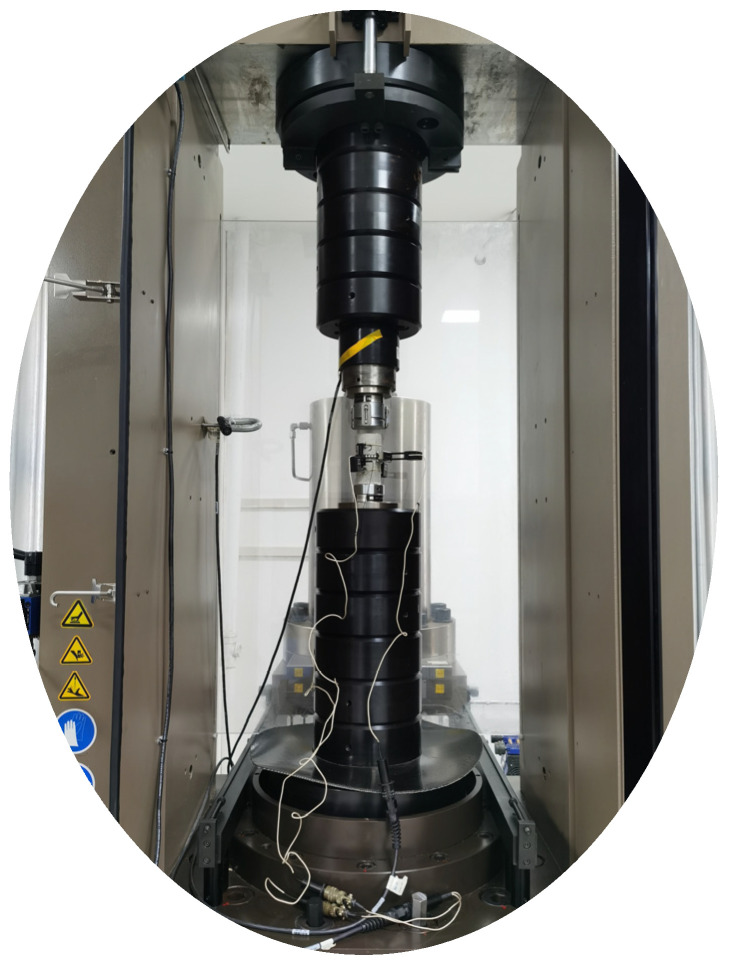
Uniaxial compression test.

**Figure 4:**
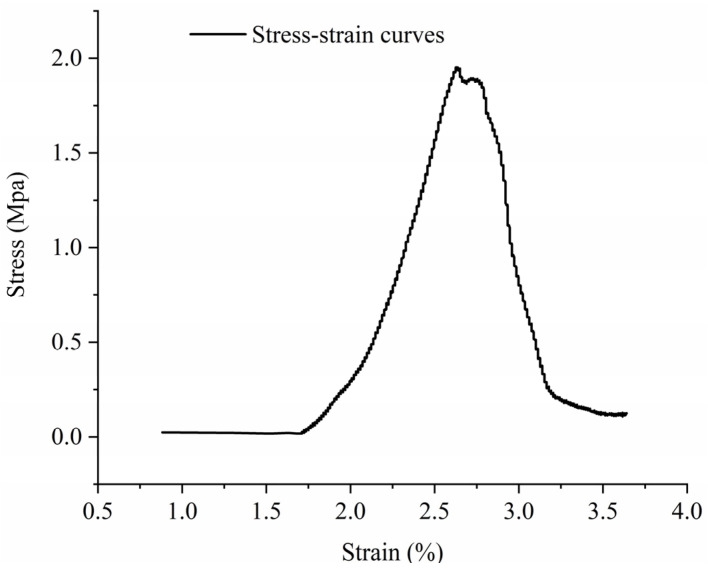
Stress–strain curve of the specimen.

A uniaxial compression test was conducted on a standard rock sample with dimensions of 100 mm in height and 50 mm in diameter. The test was executed using a rigid testing machine provided by MTS Systems, USA, as depicted in Figure 3. The specimen’s axial strain and vertical stress under graded loading were precisely recorded, and the subsequent stress–strain curve (σ–ε curve) facilitated the determination of the modulus of elasticity, E. As a representative example, the data curve for specimen No. 3 is illustrated in Figure 4.

### 3.3. Double-Sided Shear Test

**Figure 5:**
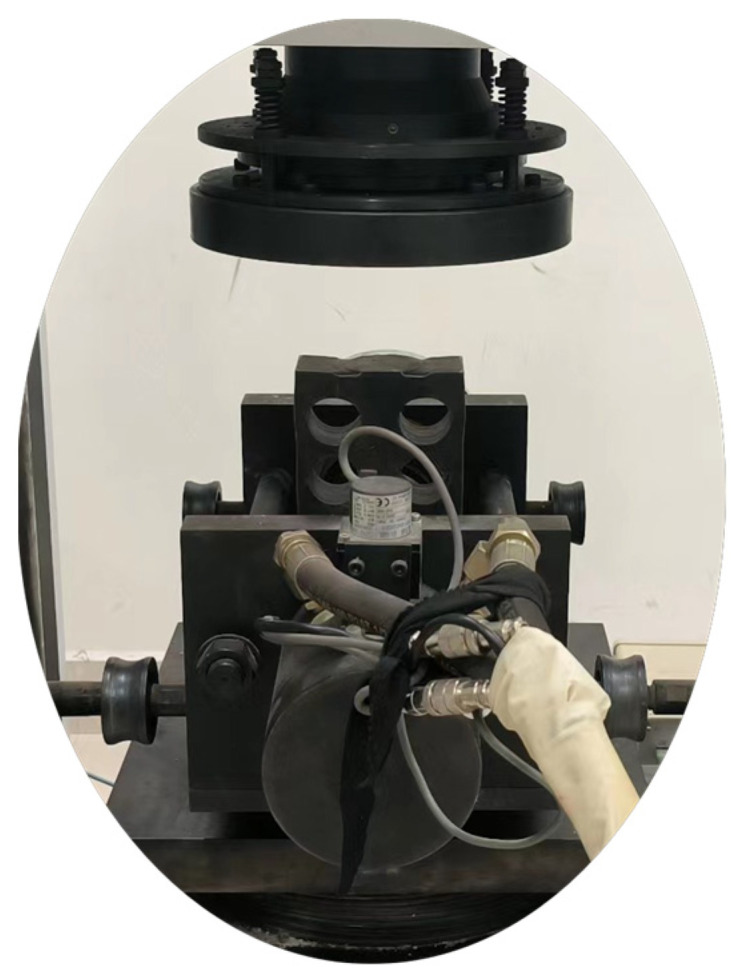
Double-sided shear meter.

**Figure 6:**
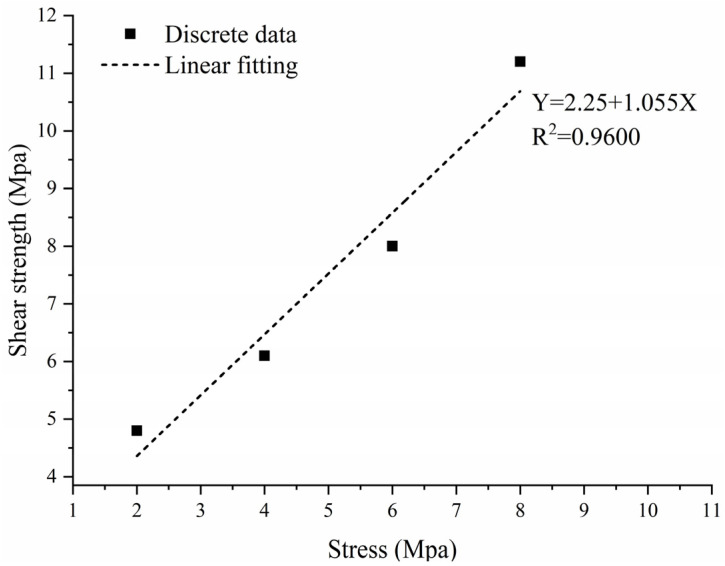
Typical σ–τ relationship curve (in MPa).

The conditioned standard specimens were positioned on the double-sided shear apparatus, as illustrated in Figure 5. For each group, five specimens underwent shearing at a rate of 0.8 mm/min under vertical pressures of 100 kPa, 200 kPa, 300 kPa, and 400 kPa, ensuring the shearing process was completed within 3–5 min. The peak shear force for each vertical loading level was recorded. The values for c and φ for the specimen group were determined via linear fitting in line with Coulomb’s law. As an illustrative point, the data curve corresponding to specimen #20 is presented in Figure 6.

### 3.4. Brazilian Cleavage Test

**Figure 7:**
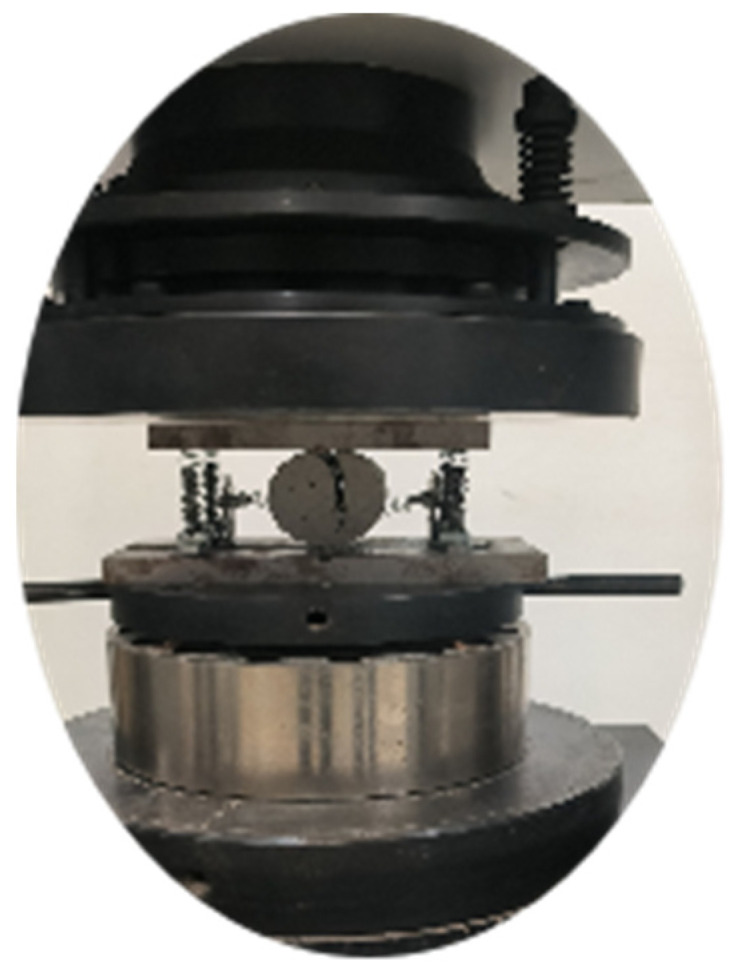
Splitting test device.

The splitting test was conducted on a specimen measuring 50 mm in both diameter and height, utilizing a universal testing machine as depicted in Figure 7. The tensile strength of the specimen was determined based on the splitting principle.

## 4. Analysis of Test Results

Physical and mechanical properties—including density, compressive strength, tensile strength, modulus of elasticity, Poisson’s ratio, angle of internal friction, and cohesion—were determined for 25 distinct material specimen groups with varying proportions. These properties were ascertained through weight measurements and the execution of uniaxial compression, splitting, and double-sided shear tests, as delineated in Table 3.

Upon analyzing the test results from specimens constructed using the above 25 ratios, the following observations were made: The density of analogous materials ranged from 2.38 to 2.93 g/cm^3^. Compressive strength varied between 0.250 and 3.725 MPa. Tensile strength spanned from 0.021 to 0.225 MPa. The modulus of elasticity oscillated between 32.23 and 389.415 MPa. The angle of internal friction had a range of 16.68 to 39.29°. Cohesion values were distributed between 0.105 and 0.717 MPa. Notably, the cohesive force distribution, extending from 0.105 MPa to 0.717 MPa, adequately covered the range requisite for most rock material model tests. The varying densities, moduli of elasticity, strengths (both compressive and tensile), cohesion, and angles of internal friction of these analogous materials can be attributed to the varying proportions of individual material components and the material’s inherent heterogeneity. While the volume of testing in this investigation remained constrained, the insights gleaned prove valuable. Concurrently, the orthogonal test results (refer to Table 3) facilitated the selection of material ratios that either aligned with or approximated the demands of analogous materials based on requisite physical and mechanical parameters for a model test.

## 5. Sensitivity Analysis of Each Factor

Sensitivity analysis constitutes a technique for uncertainty assessment, quantitatively evaluating how variations in pertinent factors influence a primary indicator or a set of such indicators. Fundamentally, this method involves the sequential alteration of associated variables to elucidate the patterns of primary indicators impacted by these factor changes. For rock masses, sensitivity analyses of physico–mechanical parameters of analogous materials offer a rapid and precise means of determining material ratios essential for model tests. A direct analytical approach encompasses the evaluation of factors through their maximum differential, wherein this differential magnitude mirrors the impact of varied factor levels on the chosen indicator. Drawing from orthogonal testing theory, each factor is averaged at a consistent level, with the extreme differential derived from the difference between the maximal and minimal values across all levels. A pronounced extreme difference suggests that varying levels of the factor yield a substantial disparity, marking it as a pivotal factor with notable influence on test outcomes. Subsequently presented is a sensitivity analysis detailing the influence of each factor on the material’s physical and mechanical properties, conducted via an extreme differential analysis.

### 5.1. Sensitivity Analysis of Density Influencing Factors

Table 4 presents the outcomes of the orthogonal test. From the data, it is evident that factor A ((iron + barite powder)/aggregate) exhibited the most significant extreme difference, succeeded by factor D (gypsum content), followed by factors B (iron powder/(iron + barite powder)) and C (binder concentration), respectively. The sequence of sensitivity of each factor to density descends in the order: A, D, B, then C. This underscores the predominant influence of factor A on density regulation.

In this study, the aggregates for the similar material comprised iron powder, barite powder, and quartz sand. Among these, iron powder and barite powder exhibited a higher specific gravity compared to quartz sand. Gypsum served as an additive, while the alcohol rosin solution, possessing a significantly lower specific gravity relative to the aggregates and gypsum, functioned as a binder. A closer examination reveals that the iron powder, barite powder, and gypsum displayed the attributes of a large mesh size and pronounced specific gravity, with quartz sand ranking second, and the alcohol rosin solution exhibiting the lowest specific gravity. Consequently, the impact on the material’s density, in descending order of significance, is the following: factor A, factor D, factor B, then factor C.

**Figure 8:**
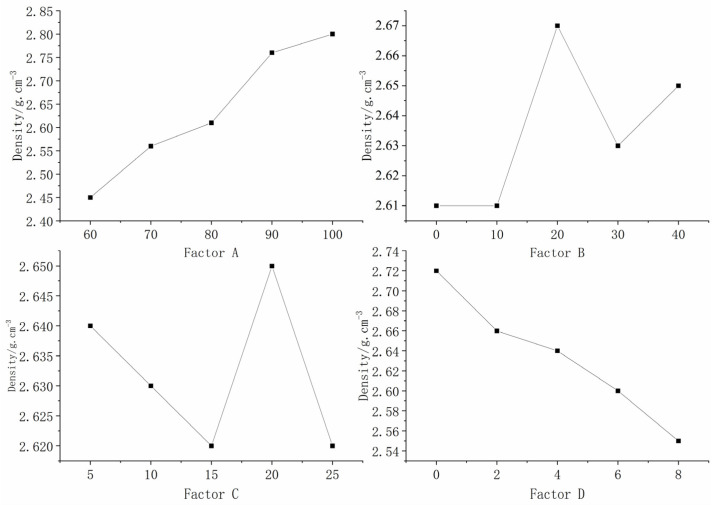
Sensitivity analysis of the factors affecting density.

To provide a clearer representation of how each factor affected density, a visual analysis chart, derived from Table 4, is presented in Figure 8. This chart elucidates that as the combined amount of iron powder and barite powder in the aggregate rose, the specimen’s density increased, whereas an increase in the gypsum content led to a reduction in density. The correlation between the remaining factors and the specimen’s density is not readily discernible.

### 5.2. Sensitivity Analysis of Factors Influencing the Compressive Strength

**Figure 9:**
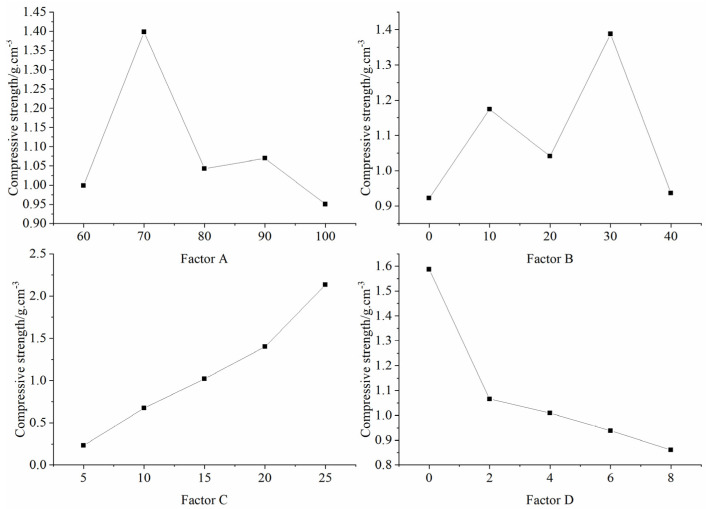
Sensitivity analysis of the factors affecting compressive strength.

Table 5 presents the results of the orthogonal tests, detailing both the mean and the extreme difference of each factor at various levels that impacted the compressive strength of the specimens. The hierarchy of influence on the compressive strength from the various factors is sequenced as C, D, B, and A, highlighting the predominant role of factor C (binder concentration) in modulating the compressive strength of the specimens. Figure 9 graphically illustrates the influence of each factor on the compressive strength, as enumerated in Table 5. The chart emphasizes that the specimen’s compressive strength augmented with an escalation in binder concentration, whereas it diminished with an elevated gypsum content. The correlation between other factors and the specimen’s compressive strength remained indistinct.

### 5.3. Sensitivity Analysis of Factors Influencing the Tensile Strength

**Figure 10:**
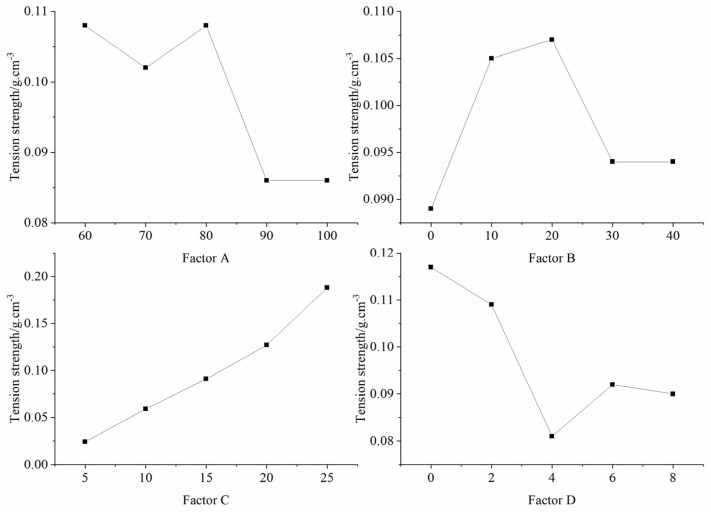
Sensitivity analysis of the factors affecting tensile strength.

Table 6 presents the outcomes of the orthogonal tests, detailing both the mean and extreme differences of each factor at varying levels that impacted the tensile strength of the specimens. Notably, factor C (binder concentration) manifested the most pronounced extreme difference, succeeded by factor D (gypsum content), factor A ((iron powder + barite powder)/aggregate), and finally factor B (iron powder/(iron powder + barite powder)). This underscores the significance of binder concentration in modulating the tensile strength of the specimens. Figure 10 graphically illustrates the influence of each factor on tensile strength, as derived from Table 6. The chart delineates that the tensile strength of the specimen augmented with an escalation in binder concentration and initially diminished, but subsequently elevated with an increase in gypsum content. The correlation between the other factors and the specimen’s tensile strength remains elusive.

### 5.4. Sensitivity Analysis of Factors Influencing the Modulus of Elasticity

**Figure 11:**
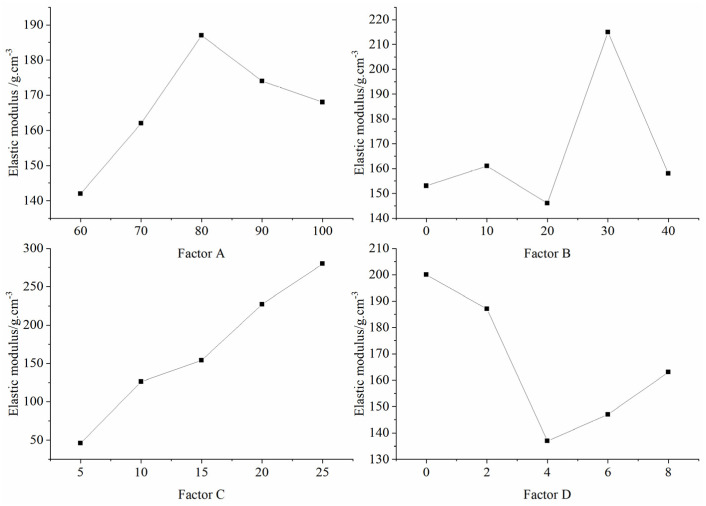
Sensitivity analysis of the factors affecting the elastic modulus.

Table 7 presents the outcomes from the orthogonal tests, detailing the mean and extreme differences of each factor at various levels that influenced the modulus of elasticity of the specimens. Notably, the sequence of influence on the modulus of elasticity is denoted by factors C, B, D, and A, underscoring the prominence of factor C (binder concentration) in modulating the modulus of elasticity. Figure 11 offers a graphical representation of these effects based on the data in Table 7. The chart delineates that an escalation in binder concentration correlated with an increase in the modulus of elasticity for the specimens. The associations between the remaining factors and the modulus of elasticity remain elusive.

### 5.5. Sensitivity Analysis of Factors Influencing the Angle of Internal Friction

**Figure 12:**
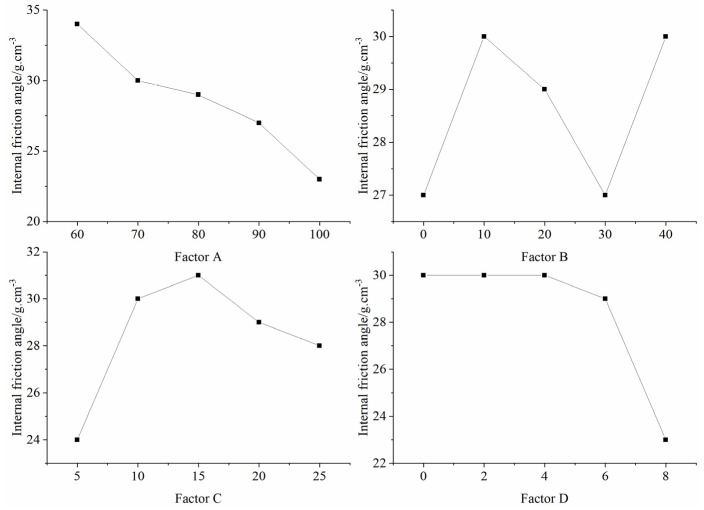
Sensitivity analysis of the factors affecting the internal friction angle.

Table 8 presents the outcomes of the orthogonal test, detailing the mean and extreme differences of each factor at different levels that influenced the internal friction angle of the specimens. Notably, the sequence of influence on the internal friction angle is denoted by factors A, D, C, and B. This suggests that factor A ((iron powder + barite powder)/aggregate) predominantly determined the internal friction angle of the specimens, as illustrated in Figure 12.

### 5.6. Sensitivity Analysis of Factors Influencing Cohesion

Table 9 presents the outcomes of the orthogonal test, detailing the mean and extreme deviations of each factor at different levels influencing the cohesion of the specimens. Notably, factor C (binder concentration) exhibited the most pronounced difference, succeeded by factors A ((iron powder + barite powder)/aggregate) and D (gypsum content), with factor B (iron powder/(iron powder + barite powder)) showing the least variation. Evidently, the binder concentration exerted a paramount influence on the specimen’s cohesion.

**Figure 13:**
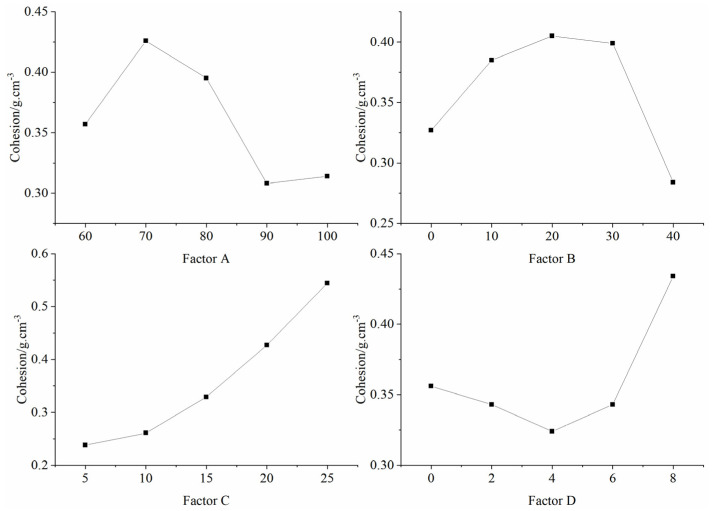
Sensitivity analysis of the factors affecting cohesion.

A visual representation of each factor’s effect on cohesion is illustrated in Figure 13, based on Table 9. The chart underscores the positive correlation between the binder concentration and specimen cohesion, while the impact of other factors remains indistinct.

From the sensitivity analysis pertaining to the influencing factors of the analogous materials, key determinants for each mechanical parameter were identified and incorporated into an equation. Specifically, the parameters are denoted as the following: *X*_1_ for (iron powder + barite powder/aggregate), *X*_2_ for iron powder/(iron powder + barite powder), *X*_3_ for binder concentration, and *X*_4_ for the total gypsum content. The regression equation for density is represented by *Y*_1_; the modulus of elasticity, cohesion, and angle of internal friction are symbolized by *Y*_2_, *Y*_3_, and *Y*_4_, respectively. Empirical formulas were derived by employing IBM SPSS Statistics v26 software for multiple regression analyses, referencing the test values of analogous material mechanical parameters listed in Table 3:

$$Y_{1}=0.009X_{1}+0.001X_{2}+0X_{3}-0.02X_{4}+1.988$$

$$Y_{2}=0.637X_{1}+0.638X_{2}+11.402X_{3}-5.833X_{4}-44.201,$$

$$Y_{3}=-0.002X_{1}-0.001X_{2}+0.016X_{3}+0.008X_{4}+0.274,$$

$$Y_{4}=-0.254X_{1}+0.044X_{2}+0.14X_{3}-0.767X_{4}+49.43.$$

**Figure 14:**
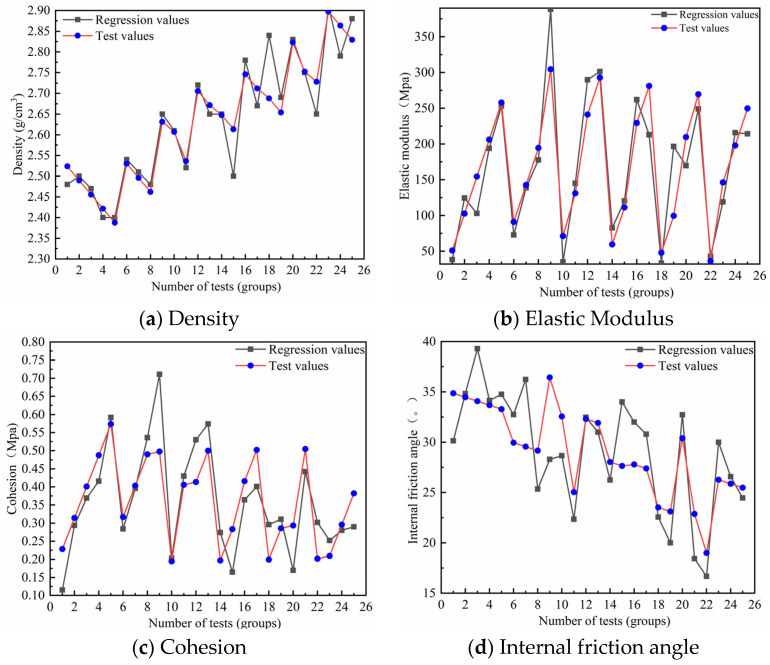
Comparison of test values and regression values.

Regression outcomes for the density, modulus of elasticity, cohesion, and angle of internal friction were derived by inputting the respective factor values from the test into the empirical equations. These outcomes were then juxtaposed with the test values, as illustrated in Figure 14.

Figure 14 reveals that the cohesion test values for test numbers 9 and 12 exhibited deviations from the regression values of 28.2% and 25.3%, respectively, surpassing the specification’s acceptable deviation threshold of 20%. Such discrepancies might have arisen from significant variations in the pore ratio due to manual mixing, air intrusion during the uniaxial test altering the pore ratio, or other unpredictable factors. For test number 9, the discrepancy between the test and regression values for the internal friction angle reached 36.4%. Given these observations, it is posited that the pronounced deviation in test number 9 stemmed from errors during specimen fabrication. While the empirical equations for both cohesion and the internal friction angle might not have aligned perfectly with test outcomes, the obtained data satisfied the criteria for simulating genuine rock masses using analogous materials. Discrepancies for the angle of internal friction test values relative to empirical values in other sequences remained below 20%, adhering to the specification guidelines. Moreover, deviations between test values for the modulus of elasticity and density from the regression values stayed under 20%. This suggests that the regression equation adequately represented the relationship between the index factors of analogous materials and both the modulus of elasticity and density of the emulated rock mass.

Upon determining the varied test requirements for physical and mechanical parameters, similar material ratios were derived based on the solution of the joint system of equations through multiple regression analysis. These ratios were then employed for the production of rock materials in geological engineering physical model tests.

## 6. Discussion

This study presents certain limitations. While the rock mass brittle damage analogue developed herein demonstrated improved brittle damage characteristics during the uniaxial compression test, its efficacy in large-scale rock mass engineering simulation remains to be validated through extensive model testing. Notably, natural rock formations exhibit non-homogeneity and anisotropy, containing internal fissures, sometimes leading to considerable fragmentation. Conversely, the current rock analogue materials are predominantly isotropic, homogeneous, and continuous. To more accurately emulate the engineering properties of genuine rock formations, further advancements in rock analogue materials are warranted. Throughout the experimental assessment of the analogous materials’ mechanical properties, temporal effects, particularly creep similarity, were overlooked. In real-world scenarios, the creep effect of rock formations can be pronounced, as observed during tunnel excavation, where deformation of exposed rock increases with excavation progression. Future studies should address this aspect, incorporating creep similarity where pertinent. Despite these limitations, the 25-material proportioning scheme remains invaluable, encompassing the physico–mechanical parameters of a vast majority of rock formations encountered in engineering geology. This schema serves as a robust foundation and reference for material selection in upcoming large-scale physical model tests.

## 7. Results and Conclusions

(1) The distribution range of physical and mechanical parameters for similar materials with varying mixing ratios is expansive. This diversity can satisfy the requirements of model tests for different rock properties, serving as a foundation for the selection of analogous materials in subsequent model tests.
(2) The influence of each factor on the physical and mechanical parameters of the material was assessed using the extreme difference analysis method. The combination of iron powder and barite powder relative to the aggregate predominantly controlled the density and internal friction angle of the specimens. Simultaneously, binder concentration was instrumental in governing the compressive strength, tensile strength, elastic modulus, and cohesion of the specimens. Further, the role of each factor on the parameters of analogous materials was scrutinized, offering insights for the selection and proportioning of materials for shaker tests. Moreover, the validity and precision of the sensitivity analysis results pertaining to the material’s mechanical parameters were reinforced through multiple regression analysis.
(3) Sensitivity analysis of the test outcomes facilitated the identification of critical factors influencing the mechanical parameters of analogous materials. These factors were then subjected to a multiple regression analysis, resulting in the formulation and validation of regression equations for material proportions. Consequently, the paper’s primary research objective—finding the optimal mixing ratio for similar materials in physical modelling tests—was achieved.
(4) The findings of this research not only offer directions for material selection in shaking table physical model tests within the realms of geotechnical and geological engineering but also guide the choice of materials in materials engineering and civil engineering model tests.

## Figures and Tables

**Table 1:** Orthogonal designs for similar materials (in %).

Level Number of Group	A	B	C	D
1	60	0	5	0
2	70	10	10	2
3	80	20	15	4
4	90	30	20	6
5	100	40	25	8

Note: The total mass of iron powder, barite powder, quartz sand, gypsum and rosin was 1000 g. The mass of alcohol required for mixing was 50 g.

**Table 2:** Similar material proportioning schemes (in %).

Level Number of Group	A	B	C	D
1	60	0	5	0
2	60	10	10	2
3	60	20	15	4
4	60	30	20	6
5	60	40	25	8
6	70	0	10	4
7	70	10	15	6
8	70	20	20	8
9	70	30	25	0
10	70	40	5	2
11	80	0	15	8
12	80	10	20	0
13	80	20	25	2
14	80	30	5	4
15	80	40	10	6
16	90	0	20	2
17	90	10	25	4
18	90	20	5	6
19	90	30	10	8
20	90	40	15	0
21	100	0	25	6
22	100	10	5	8
23	100	20	10	0
24	100	30	15	2
25	100	40	20	4

**Table 3:** Orthogonal experiment results of similar material ratios.

Level Number of Group	Density/g.cm^−3^	Compresive Strength/Mpa	Tension Strength/Mpa	Elastic Modulus/Mpa	Poisson’s Ratio	Internal Friction Angle	Cohesion/Mpa
1	2.46	0.25	0.021	36.137	0.21	30.14	0.105
2	2.5	0.853	0.099	124.46	0.12	34.82	0.194
3	2.36	1.05	0.102	102.94	0.15	39.29	0.369
4	2.38	1.334	0.123	193.909	0.21	34.14	0.416
5	2.4	1.496	0.196	253.132	0.12	34.76	0.592
6	2.54	0.677	0.052	72.872	0.09	32.74	0.284
7	2.55	1.076	0.094	138.735	0.14	36.23	0.396
8	2.48	1.238	0.12	177.707	0.06	25.34	0.536
9	2.65	3.725	0.216	389.415	0.2	28.3	0.717
10	2.61	0.266	0.027	34.984	0.04	28.66	0.203
11	2.52	0.727	0.075	144.948	0.05	22.35	0.430
12	2.72	1.895	0.174	289.777	0.23	32.47	0.510
13	2.63	1.935	0.225	301.302	0.13	31	0.574
14	2.65	0.238	0.022	82.67	0.15	26.24	0.274
15	2.5	0.418	0.044	115.352	0.22	34	0.165
16	2.78	1.31	0.122	262.076	0.15	32	0.364
17	2.67	1.865	0.133	212.958	0.07	28.8	0.401
18	2.84	0.226	0.021	32.23	0.06	22.56	0.296
19	2.69	0.668	0.037	176.464	0.19	20	0.311
20	2.83	1.283	0.109	169.621	0.13	32.72	0.170
21	2.75	1.634	0.173	248.991	0.28	18.43	0.432
22	2.65	0.171	0.023	42.847	0.17	16.68	0.302
23	2.93	0.758	0.065	119.047	0.20	28.91	0.252
24	2.72	0.964	0.075	210.836	0.13	26.58	0.270
25	2.88	0.096	0.096	214.209	0.13	23.42	0.280

**Table 4:** Analysis of density extremes (in g/cm^−3^).

Level Number of Group	A	B	C	D
1	2.45	2.61	2.64	2.72
2	2.56	2.61	2.63	2.66
3	2.61	2.67	2.62	2.64
4	2.76	2.63	2.65	2.60
5	2.80	2.65	2.62	2.55
Extreme deviations	0.35	0.06	0.03	0.17

**Table 5:** Analysis of extreme differences in compressive strength (in Mpa).

Level Number of Group	A	B	C	D
1	0.999	0.922	0.233	1.587
2	1.398	1.174	0.675	1.066
3	1.043	1.041	1.020	1.009
4	1.070	1.388	1.399	0.938
5	0.950	0.936	2.133	0.861
Extreme deviations	0.448	0.466	1.900	0.725

**Table 6:** Analysis of extreme differences in tensile strength (in MPa).

Level Number of Group	A	B	C	D
1	0.108	0.089	0.024	0.117
2	0.102	0.105	0.059	0.109
3	0.108	0.107	0.091	0.081
4	0.086	0.094	0.127	0.092
5	0.086	0.094	0.188	0.09
Extreme deviations	0.023	0.019	0.164	0.036

**Table 7:** Analysis of extreme differences in modulus of elasticity (in MPa).

Level Number of Group	A	B	C	D
1	142.515	153.405	46.363	200.999
2	162.543	161.755	126.639	187.732
3	187.810	146.834	154.416	137.130
4	174.859	215.459	227.536	147.032
5	168.186	158.460	280.959	163.020
Extreme deviations	45.294	68.625	234.597	63.869

**Table 8:** Analysis of the extreme difference in the angle of internal friction (in °).

Level Number of Group	A	B	C	D
1	34.630	27.132	24.856	30.726
2	30.254	30.200	30.309	30.612
3	29.213	29.636	31.435	30.705
4	27.617	27.052	29.682	29.072
5	23.229	30.922	28.660	23.826
Extreme deviations	7.013	3.870	6.579	6.900

**Table 9:** Extremum differential analysis of cohesion (in MPa).

Level Number of Group	A	B	C	D
1	0.357	0.327	0.238	0.356
2	0.426	0.385	0.261	0.343
3	0.395	0.405	0.329	0.324
4	0.308	0.399	0.427	0.343
5	0.314	0.284	0.544	0.434
Extreme deviations	0.037	−0.007	0.306	0.032

## Data Availability

All data and materials support our published claims and comply with field standards. The datasets generated and/or analyzed during the current study are not publicly available. All software applications or custom codes support our published claims and comply with field standards.
